# Synthesis of Ni^2+^-functionalized polydopamine magnetic beads for facilitated purification of histidine-tagged proteins

**DOI:** 10.1186/s13568-023-01613-z

**Published:** 2023-10-13

**Authors:** Alireza Shariati, Sara Ali Hosseinzadeh, Zahra Barghi, Sogand Sadat Mortazavi, Kosar Atarod, Fatemeh Sadat Shariati, Behrokh Farahmand

**Affiliations:** 1 Department of Materials Engineering, Tarbiat Modares University, P.O. Box 14115-143, Tehran, Iran; 2 Department of Nanobiotechnology, New Technologies Research Group, Pasteur Institute of Iran, Tehran, Iran; 3 Infuenza Research Lab, Pasteur Institute of Iran, Tehran, Iran; 4 Farzanegan high school, National Organization for Development of Exceptional Talents, Tehran, Iran

**Keywords:** Magnetic beads, Facillated purification, Polydopamine, His-tagged protein, Recombinant protein

## Abstract

Facilitated purification of proteins, at a low cost and a short time, is one of the key steps in the industrial production of recombinant proteins. In the current study, polydopamine nanoparticles (PDA-NPs) are considered in the synthesis of magnetic beads for purifying recombinant proteins due to advantages such as biocompatibility/ biodegradability, easy synthesis, as well as the ability to directly chelate metal ions. They were synthesized in Tris buffer (pH: 8:5), then chelated with Fe^3+^(20 mg) and Ni^2+^ ions at concentrations of 2, 3, 5, and 7 mg/ml. Prepared nanoparticles were characterized through scanning electron microscopy (SEM), ultraviolet-visible spectroscopy (UV-vis), dynamic light scattering (DLS), Inductively Coupled Plasma (ICP), and vibrating sample magnetometer (VSM). The size distribution of the particles was reported in the narrow range of 120–140 nm and 200 to 220 nm by the SEM image and DLS analysis, respectively. The chelation of ions on the surface of the nanoparticle was confirmed by the ICP technique with a magnetization of 35.42 emu/g. The highest adsorption rate of Ni^2+^ ions to polydopamine was obtained at a ratio of 1.4. The SDS-PAGE and western blot analysis confirmed the purification of eGFP and Hsp40 by PDA/Fe^3+^/Ni^2+^ at 26 and 40 kDa compared to the commercial nickel column. Moreover, the concentration of purified eGFP by PDA/Fe^3+^/Ni^2+^ was reported 138.83 µg/ml by the fluorescent signals, which is almost equal to or more than the protein purified by commercial Ni-NTA column (108.28 µg/ ml). The stability of PDA/Fe^3+^/Ni^2+^ has also been evaluated by ICP-OES for 10 days, and the result suggested that PDA magnetic beads were stable. Therefore, it can be concluded that PDA/Fe^3+^/Ni^2+^ have the ability to purify recombinant proteins in one less step and shorter time.

## Introduction

Facilitated separation and purification of recombinant proteins, at a low cost, in a short time, and without the need for multiple steps of synthesis of magnetic beads is one of the key steps in the industrial production of recombinant pharmaceutical proteins(Ghosh 2002; Wang et al. 2020). Common methods for the purification of recombinant proteins in the biotechnology industry include hydrophobic chromatography, ultrafiltration, ion exchange chromatography, gel filtration chromatography, and affinity chromatography, which is selected according to the properties of the protein and the separation requirements (Zhou et al. 2021). In affinity chromatography techniques, protein labels play an essential role in the purification of recombinant proteins. The labeling technology has many advantages, such as (1) single-step separation and purification, (2) achieving higher protein activity, and (3) good versatility and usability for different proteins. Hexa-histidine can be the most popular label for conjugating recombinant proteins, and is used as the most widely used affinity label due to its relatively low molecular weight, low immunogenicity, and hydrophilic properties (Hage et al. 2012; Turková 1978; Yang et al. 2015). In addition, histidine tag is available at different vectors and hosts for protein expression, which conjugate at the end of the N-terminal or C-terminal of protein(Aslantas and Surmeli 2019). However, the purification method by immobilized metal affinity (IMAC) has many drawbacks such as long and complex steps that take a long time to purify proteins (Liao et al. 2013; Russell et al. 2005; Zhou et al. 2021). Recombinant proteins are typically engineered and labeled with histidine, this expressed protein in host cell extracts is attached and purified by nickel, cobalt, copper, or zinc using the principles of affinity at several stages(Salimi et al. 2017; Xie et al. 2010). Therefore, due to the fact that the market for recombinant pharmaceutical products is growing at a high rate, and the production process of these products is becoming more and more prosperous, the facilitated purification of proteins by magnetic nanomaterials has attracted a great deal of attention in the purification process of recombinant proteins. The use of these magnetic materials leads to high yield, simplifies the purification process, and effectively shortens the time of the purification process by magnetic separation of histidine-labeled proteins in crude biological samples (an external magnet)(Sahu et al. 2011; Yang et al. 2015). In addition, magnetic nanomaterials have a specific surface area that allows efficient adsorption of ions on their surface, therefore can improve the adsorption of proteins to ions, and simplifies the separation of the target protein in a sample with very low concentration sample(Salimi et al. 2017; Wang et al. 2020). For example, Tural et al. immobilized the histidine-labeled recombinant benzaldehyde lyase on an epoxy magnetic backup(Tural et al. 2013). Ho et al. immobilized the histidine-labeled protein using a Cu^2+^-immobilized acetic acid-modified carrier(Ho et al. 2004). Yan et al. purified phosphorylated polypeptides using polydopamine with Ti^2+^(Yan et al. 2013). Shin et al. synthesized shell-core nanoparticles (Fe_3_O_4_ @ nickel–silicate) to immobilize histidine-labeled enzymes. In this study, Fe_3_O_4_ was encapsulated in a nickel-silicate coating, and the results showed the high binding capacity for tobacco etch virus protease enzyme (TEV), which is easily separated by an external magnetic field(Shin et al. 2016). Zhou synthesized NiFe_2_O_4_ magnetic nanoparticles for the purification of recombinant histidine-labeled glutamate dehydrogenase with immobilization and one-step affinity, the result showed that synthesized magnetic nanoparticles have better binding strength than commercial Ni-NTA resin(Zhou et al. 2021). Park et al. investigated the immobilization of histidine-labeled GFP proteins with cationic ferrite magnetic nanoparticles. In this study, four types of magnetic nanoparticles were prepared cobalt- iron oxide, copper-iron oxide, nickel-iron oxide, and iron oxide III (as negative control). The results showed that among these nanoparticles, cobalt-iron oxide is the most effective immobilizer. Also, the research showed that among different nanoparticles, dopamine due to its abundant indole amine and catechol groups has good adhesion to bind to divalent metal ions directly (Park et al. 2018). Thus, in the current study, the magnetic beads based on PDA NPs were synthesized with a dual-capacity ionic coating of Ni^2+^ and Fe^3+^ for the purification of recombinant proteins in a short time, with low cost, and a simple and facilitated process of synthesis (without the need for multiple steps). This process enables facilitated purification of proteins in the shortest time based on direct chelation of PDA NPs with dual ions Ni^2+^ and Fe ^3+^ ions.

## Experimental sections

### Materials and characterizations

Iron chloride (FeCl_3_), Dopamine hydrochloride, Tris-HCl, and all other chemicals were purchased from Sigma-Aldrich (USA), and used without further treatment. A stirrer (IKA Company, Germany) was used to synthesize the PDA/Fe^3+^/Ni^2+^ nanoparticles. The morphology and size of the PDA/Fe^3+^/Ni^2+^ nanoparticles were investigated by scanning electron microscopy (SEM) (MIRA3, Tescan, Czech Republic) and Image J software. To determine the adsorption and release of Fe^3+^ and Ni^2+^ ions by polydopamine nanoparticles, an inductively coupled plasma (ICP-OES) VISTA-PRO model was used. In addition, dynamic light scattering (DLS) (Zetasizer Nano, Malvern, England) was used to measure the hydrodynamic size of particles. Magnetic characteristics of PDA/Fe^3+^/Ni^2+^ were measured by a vibrating sample magnetometer (VSM, Model MDKB) at 25 °C. The presence of the metallic elements within the magnetic beads was investigated using XRD (X-ray Diffractometer: DX-2700BH ( analysis.

### Strains and plasmids

#### ***E.coli*** strains, plasmids, and growth conditions

*Escherichia coli* DH5α and *E. coli* BL21 (DE3) transformed with pEGFP plasmid (Addgene, USA) and pET21a-*hsp40* (in our pervious study(Shariati et al. 2021) for plasmid propagation, and gene expression, respectively. The strains were obtained from the cell bank of the Pasteur Institute of Iran. The transformed bacteria via pET28a-*egfp* and pET21a-*hsp40* were cultured aerobically in Luria Bertani (LB) broth supplemented with the appropriate antibiotics (35 µg/mL kanamycin and 100 µg/mL ampicillin) at 37 ºC(Shariati et al. 2021).

#### eGFP and Hsp40 expression in the inducible system

A clone containing pET28a-*egfp* plasmid and pET21a-*hsp40* was inoculated into a 5 mL LB medium containing 35 µg/mL kanamycin and 100 µg/mL ampicillin, respectively, and allowed to grow at 37ºC, 180 rpm (to OD_600nm_ ~ 0.6). Then, bacterial culture was induced with IPTG and incubated at 37ºC, 180 rpm for 6 h. The protein expression of Hsp40 and eGFP was analyzed by the SDS-PAGE technique. Moreover, the expression of eGFP measured using fluorimetry (485 nm excitation and 528 nm emission) (fluorimeter, BioTek, USA) and standard curve “eGFP concentration vs. fluorescence intensity” with a similar method for the expression of eGFP protein in our previous study(Shariati et al. 2021).

#### Preparation of PDA nanoparticles

The PDA nanoparticles were synthesized according to the method described below. 62.105 mg of Dopamine (DA) were dispersed in 10 mL of Tris buffer (100 mM, pH 8.5), and mixed on a stirrer at room temperature for 8 h at 500 rpm. The PDA nanoparticle solution was centrifuged at 13,000 rpm for 15 min. The pellet was removed, and the supernatant was filtered through a 0.22 μm syringe filter. Then, the product was dialyzed against deionized water (DI) for 3 days using a dialysis bag (cutoff: 12,000–14,000 Da) at 500 rpm, and freeze-dried(Gu et al. 2018; Shariati et al. 2023a; Shariati et al. 2023a, b).

#### Coating of PDA with Fe^3+^ and Ni^2+^

Briefly, a solution of PDA-NPs was prepared in 5 ml of deionized water with a concentration of 1 mg/ml. 20 mg of FeCl_3_ was added to the PDA-NPs solution. Concurrently, different amounts of Ni_2_SO_4_ (2, 3, 5, and 7 mg) were dissolved in 5 ml of deionized water. Then, various weight ratios of Ni^2+^ ions per mg PDA-NPs were prepared (0.4:1, 0.6:1, 1:1, and 1.4:1). Then, the solution was stirred at 500 rpm for 7 h. The solutions containing PDA-NPs coated with Ni^2+^ /Fe^3+^ ions were filtered by a dialysis bag (cut off: 12,000–14,000 Da) against distilled water (100 cc) for 24 h to remove the unreacted ions. The number of reacted ions was investigated by ICP-OES analysis.

In this study to investigate the effect of different weight amounts of Ni^2+^ ions in the adsorption to the surface of PDA, the mg of Fe ions was considered constant (the highest amount of Fe ions). By fixing the mg of Fe ions, the effect of different weight amounts of Ni^2+^ ions on adsorption to surface of PDA was investigated.

#### Purification of GFP and Hsp40 protein by PDA/Fe^3+^/Ni^2+^ nanoparticles

500 µL of cell lysate containing green fluorescent protein (eGFP; total protein concentration of 2 mg/mL) was added to 1 ml of PDA/Fe^3+^/Ni^2+^ nanoparticles (In concentrations: 2, 5, 10, and 15 mg/mL) at phosphate-buffered saline (PBS) (pH 7.4, 30 mM). After 15 min incubation at room temperature, the solution containing magnetic nanoparticles bonded to His-Tag proteins was separated by a magnet. Then, the nonspecifically bound proteins were removed by 1 mL of PBS buffer containing 10 mM imidazole, and the His-eGFP was finally eluted by the addition of 1 mL of PBS buffer containing 300 mM imidazole. The purity of the His-eGFP was analyzed by 12% sodium dodecyl sulfate-polyacrylamide gel electrophoresis (SDS-PAGE).

To purify eGFP, 300 µL of cell lysate containing green fluorescent protein (eGFP; total protein concentration of 2 mg/mL) was centrifuged at 7000 rpm for 10 min. The pellet was resuspended in the lysis buffer (50 mM NaH_2_PO_4_.2H_2_O, 300 mM NaCl, 1 mg/ml lysozyme, pH 8) and lysed by sonication (10 pulse 2 min) on ice. The lysate was then centrifuged at 14,000 g at 4 °C for 25 min, and the supernatant was loaded on an equilibrated Ni-NTA resin (Qiagen) for 1 h at 4 °C. The resin was washed several times with the wash buffer (300 mM NaCl, 20 mM imidazole, 50 mM NaH_2_PO_4_.2H_2_O, pH 8). Finally, the protein was eluted with a buffer containing 50 mM NaH_2_PO_4_.2H_2_O, 300 mM NaCl, and 250 mM imidazole (adjusted to pH 8). The purity of purified eGFP was analyzed using gel 12% sodium dodecyl sulfate (SDS)-polyacrylamide gel electrophoresis (Shariati et al. 2022; Shariati, Keramati, Shariati et al. 2021a, b; Shariati, Norouzian, Shariati et al. 2021a, b).

Moreover, the purification of Hsp40 was performed with a similar protocol for purification of eGFP protein by PDA/ Fe^3+^/Ni^2+^ and commercial column (as Positive control). PDA/ Fe^3+^ and PDA were considered as negative control. The reusing of the synthesized beads for Hsp40 was investigated in 6 consecutive runs, and the purity of the His-eGFP was analyzed by 12% sodium dodecyl sulfate-polyacrylamide gel electrophoresis (SDS-PAGE).

#### Specificity of the PDA/ Fe^3+^/Ni^2+^ to his-tagged protein

To investigate the specificity and purity of PDA/Fe^3+^/Ni^2+^ to eGFP-His-Tag compared to purified protein by Ni-NTA columns, the synthesized magnetic bead nanoparticles were mixed with a cell lysate containing eGFP-His-Tag. The fluorescence intensity of the purified GFP protein (using PDA magnetic beads NPs) in elution 1, 2, and 3 were measured by a fluorimeter device (485 nm excitation and 528 nm emission, BioTek, USA) at three replicates, and compared to purified protein by Ni-NTA columns.

#### Stability test for PDA/ Fe^3+^/Ni^2+^

The Ni^2+^ and Fe^3+^ releases of PDA/ Fe^3+^/Ni^2+^NPs were evaluated at a temperature of 37 °C. Synthesized magnetic beads were dispersed at a concentration of 10 mg/ml at 5 ml of PBS, then dialyzed with a dialysis bag (cut off: 12,000–14,000 Da) against distilled water (100 mL) for 10 days by ICP-OES analysis(Shariati et al. 2023a, b).

**Fig. 1:**
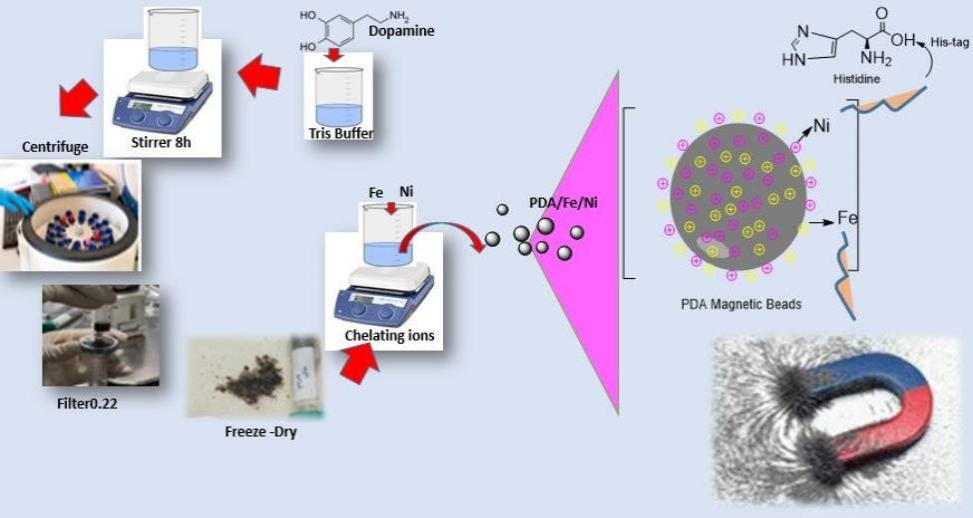
Synthesis of polydopamine nanoparticles chelated with Fe^3+^ and Ni^2+^, and its application in facilitated purification

### Statistical analysis

XRD, DLS, FTIR, VSM, and ICP-OES plots were performed by Origin Pro 2018. The polydopamine NPs diameters were determined by Image J software (https://imagej.nih.gov/ij/ index. html).

## Results

### Characterization of PDA/ Fe^3+^/Ni^2+^

#### SEM and DLS analysis

**Fig. 2:**
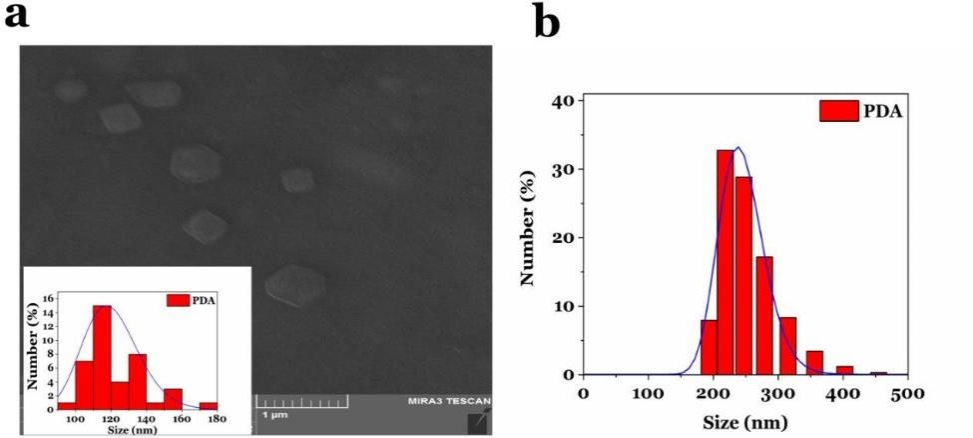
SEM analysis. (**a**) The SEM image and the average size distribution of PDA NPs. (**b**) The hydrodynamic size distribution of PDA NPs using the DLS technique

Particle size and morphology of polydopamine nanoparticles coated with Ni^2+^ and Fe^3+^ ions were determined by scanning electron microscope (SEM) (Fig. 2a). The several polydopamine nanoparticles (70 NPs) were randomly selected on the SEM image, and measured with Image J software. The diameter of the particles was obtained in a narrow range of 120–140 nm. The nanoparticles were uniform, dispersed, and spherical. In addition, the average particle diameter was measured by hydrodynamic particle distribution analysis (DLS) in the range of 200 to 220 nm (Fig. 2b). In this method, the Brownian motion of particles in the fluid phase is used to distribute the dimensions of particles in a solution.

### Results of adsorption and adsorption efficiency of Fe^3+^ and Ni^2+^ ions chelated on polydopamine

**Fig. 3:**
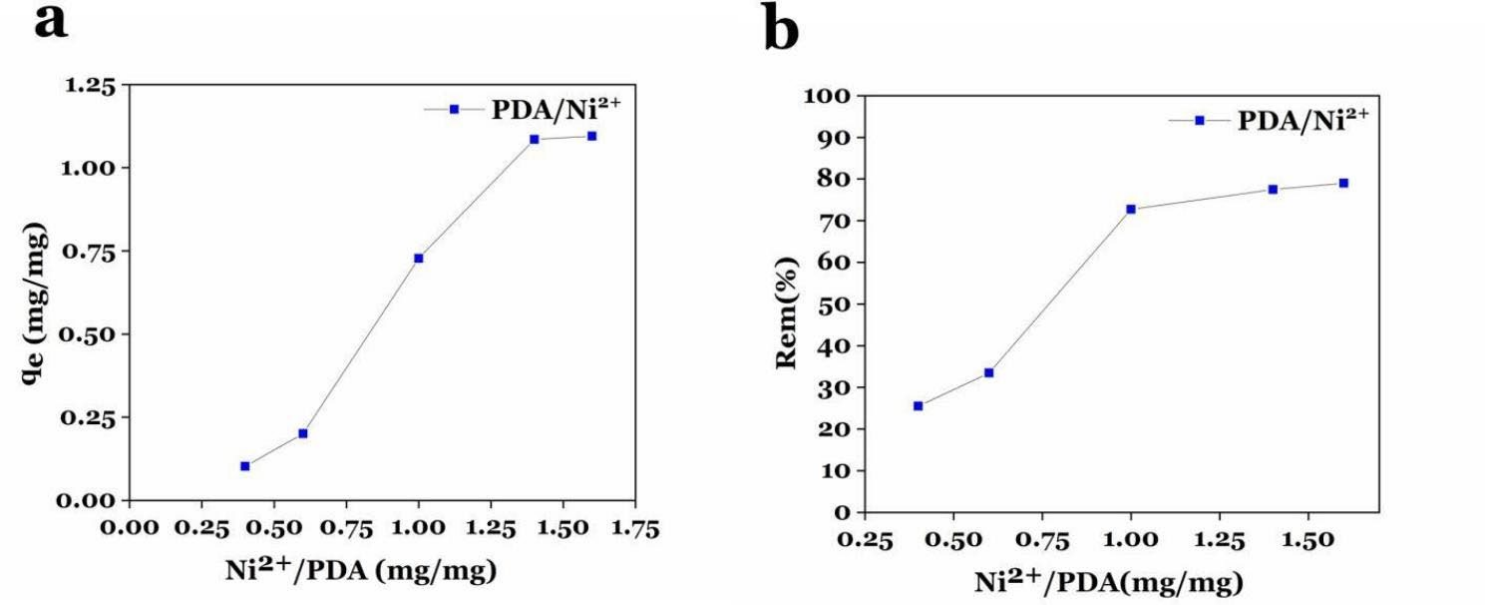
Adsorption study. (**a**) Adsorption plot (q_e_) of Ni^2+^ ions on PDA/Fe^3+^ and (**b**) Ni^2+^ ion adsorption efficiency of PDA/Fe^3+^.

In this study, the adsorption (q_e_) of Ni^2+^ ions (in different weight ratios: 0.4, 0.6, 1, and 1.4 mg/mg of ion to PDA) and Fe^3+^ ions on the surface of PDA NPs was investigated using dual ICP-OES (inductively coupled plasma emission spectrometry analysis). As shown in Fig. 3a, at the adsorption plot, with the increasing weight ratio of Ni^2+^ ion to PDA, the slope was also increased until 1.4 ratio, and then its trend is fixed. As the adsorption graph and the adsorption efficiency (REM%) plots show, ratio 1.4, in addition to having the highest ion adsorption, also has a suitable adsorption efficiency, thus, this ratio can be economical for the next steps of the preparation of magnetic beads (Fig. 3a and b).

**Fig. 4:**
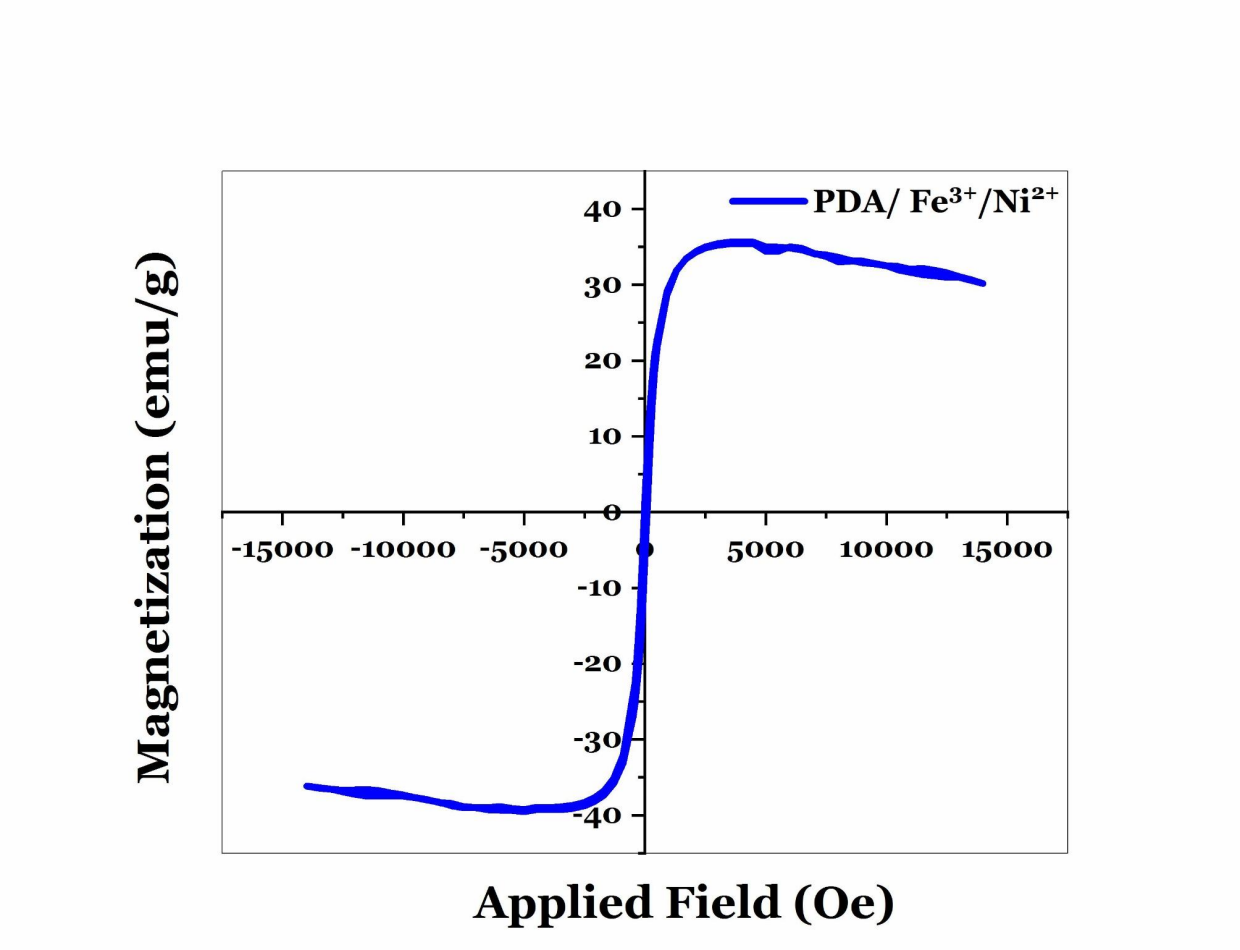
VSM plot for PDA/ Fe^3+^/Ni^2+^.

The hysteresis loop (Fig4) revealed the magnetic behavior of PDA/Ni^2+^ at room temperature after coating with Fe^3+^. The saturated magnetization (Ms) of PDA magnetic beads was reported 35.42 emu/g. Therefore, these beads have great potential for application in biotechnology for the purification of recombinant proteins.

### Purification of eGFP by PDA magnetic beads

The fluorescence intensity of the purified eGFP protein using PDA magnetic beads in elution 1, 2, and 3 were measured by a fluorimeter device at three replicate (Table 1). As shown, the concentration of purified eGFP with PDA magnetic beads was the highest at elution 2 compared to purified eGFP protein using a commercial Ni-NTA column, and measured in the range of 138.83 µg/ml. Moreover, the effect of different concentrations of magnetic beads (2, 5, 10, 15 mg/ml) on the elution of eGFP proteins was investigated by intensity of fluorescence signals. The result show that the amount of purified protein (µg/ml) based on the intensity of fluorescent signals was 85.43, 110.23, 138.83, and 139.85, respectively. Thus, with the increasing concentration of magnetic beads, the fluorescence intensity was also increased, but from the concentration of 10 mg/ml onwards, the fluorescence intensity showed a steady trend. This means that the concentration of 10 mg/ml for magnetic beads is economical in terms of material, and can be used for purification process.

**Table 1:** The amount of purified protein (µg/ml) based on the intensity of fluorescent signals

PDA/Fe^3+^/Ni^2+^ Elution 1	PDA/Fe^3+^/Ni^2+^ Elution 2	PDA/Fe^3+^/Ni^2+^ Elution3	Commerical Columns Ni-NTA
16,222 ± 105(RFU)	26,170 ± 100(RFU)	4166 ± 2.86(RFU)	20,535 ± 270(RFU)
84.94 (µg/ml)	138.83 (µg/ml)		108.28 (µg/ml)

### SDS PAGE analysis of purified eGFP and Hsp40 protein using polydopamine magnetic beads

**Fig. 5:**
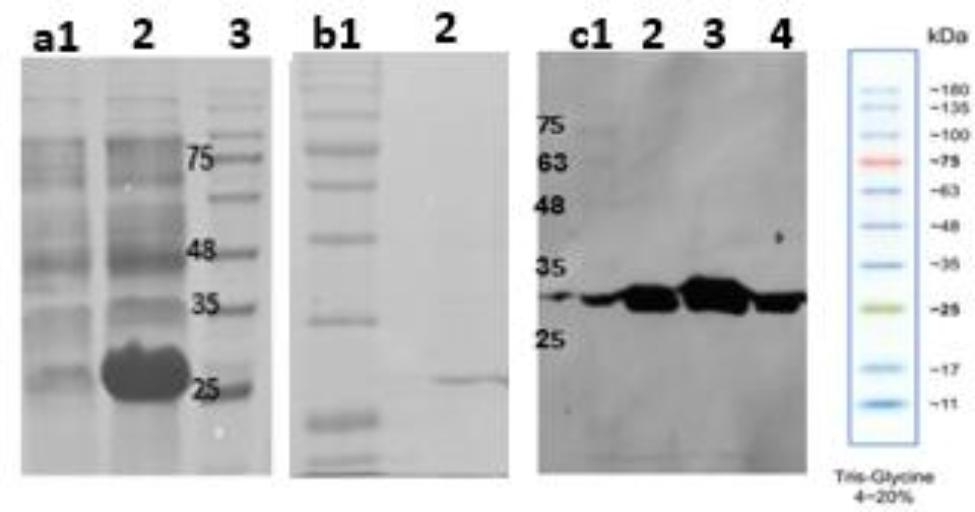
Purification analysis of eGFP protein using magnetic beads. (**a**) 12% SDS-PAGE analysis of eGFP expression [Lane 1: before induction, Lanes 2: 16 h after induction in the 5ml culture, lane 3: Protein marker], (**b**) 15% SDS-PAGE analysis of purified eGFP [Lane1: protein marker, Lane 2: purified eGFP by PDA magnetic beads in elution 2], (**c**) Western blot analysis of purified GFP protein [Lane 1: marker protein, lane 2 and 3: purified eGFP protein with PDA magnetic beads in elution 1 and 2, respectively, Lane 4: purified eGFP protein with a commercial Ni-NTA column]. The protein marker molecular weights are 180, 135, 100, 75, 63, 48, 35, 25, 17, and 11 kDa

Figure 5a **and b** show the expression of purified eGFP protein in the weight range ~ 26 K.Da in the bacterial host *E.col*BL21 (DE3). In addition, Fig. 5c demonstrates that the intensity of the protein band purified by magnetic beads in lane 3 (elution 2) is higher than in lane 2 (elution 1), and the protein purified by the commercial column in lane 4.

**Fig. 6:**
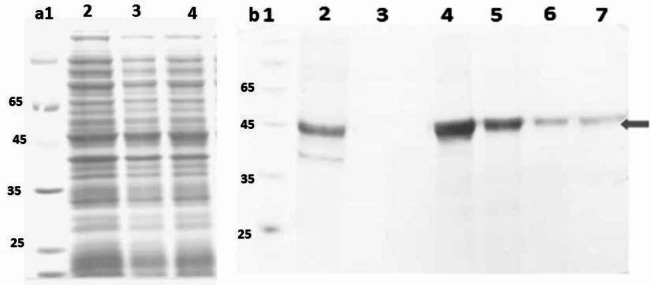
Purification analysis of Hsp40 protein by magnetic beads and commercial column (Ni-NTA). 12% SDS-PAGE analysis of Hsp40 expression (**a**) [Lane 1: The protein marker, Lanes 2, 3, and 4: Cell lysate, cell lysate after treatment by PDA, and PDA/Fe^3+^ nanoparticles (as negative control), respectively], (**b**) [ Lane 1: The protein marker, Lane 2: eluted protein by commercial column(Ni-NTA), Lane 3: eluted protein by PDA/Fe^3+^/Ni^2+^ nanoparticles in run 6, Lane 4, 5, and 6: eluted protein by PDA/Fe^3+^/Ni^2+^nanoparticles in run 1, run 2, run 4, and run 5 (reusability), respectively]

Figure 6 Shows the expression of purified Hsp40 protein in the weight range 40 K.Da in the bacterial host *E.col*BL21 (DE3) in 6 different runs compared with eluted protein by PDA/ Fe^3+^ and PDA (as negative control) and commercial column (as positive control). The result demonstrated that synthesized magnetic beads based on PDA are reusable after 5 runs of the purification process. The specificity of them remained unaffected, and its efficiency was still above 90% which is similar to developed magnetic beads by Wang etal(Wang et al. 2020). However, the result show that the amount of purified protein gradually decreases after repeated use due to the possible detaching of nickel from polydopamine nanoparticles.

### The stability study of PDA/ Fe^3+^/Ni^2+^

**Fig. 7:**
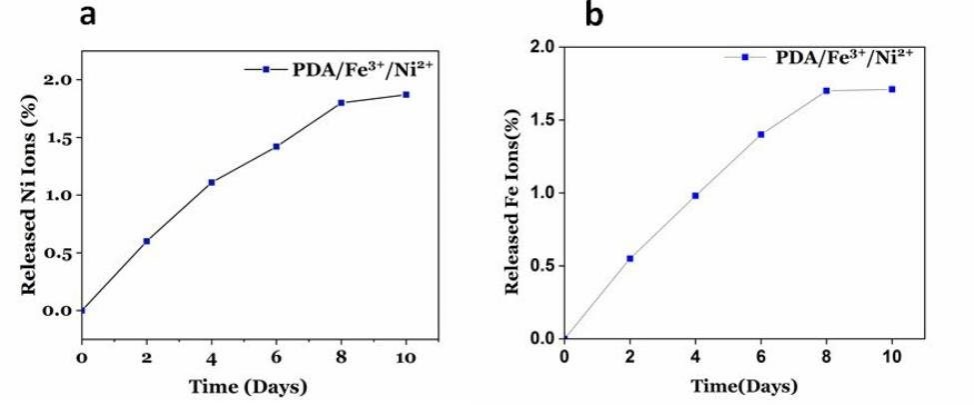
The percentage of (**a**) Ni^2+^ and, (**b**) Fe^3+^ ions released from PDA/Fe^3+^/Ni^2+^ nanoparticles in 10 days intervals

The stability of PDA/ Fe^3+^/Ni^2+^ has also been evaluated by measuring free Ni^2+^ and Fe^3+^ ions in the supernatant after filtering with a dialysis bag (cutoff: 12,000–14,000 Da) against deionized water (DI) for 10 days. The result of ICP-OES **(**Fig. 7a **and b)** illustrated ~ 2% of free Ni^2+^ions and ~ 1.7% of free Fe^3+^ in the supernatant after 10 days.

### XRD analysis

#### Response

**Fig. 8:**
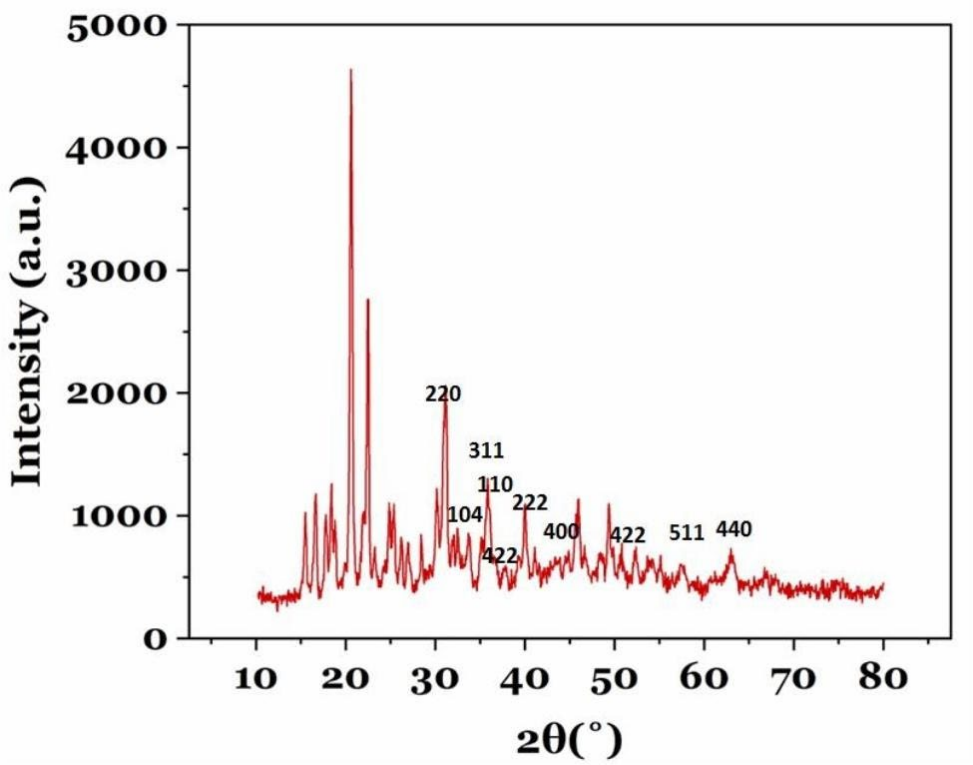
XRD pattern of PDA/ Fe^3+^/Ni^2+^ magnetic beads

XRD analysis (Fig. 1) was used to investigate the nature of Ni and Fe ions on the surface of polydopamine nanoparticles. From the results acquired by XRD analysis, it can be inferred that Fe ions on the surface of polydopamine probably have magnetite properties. In the XRD pattern, PDA /Fe^3+^/Ni^2+^nanoparticles could be indexed with the diffraction peaks of Fe_3_O_4_ and Fe_2_O_3_ which is similar to previous studies(Wang et al. 2020). XRD analysis of the developed magnetic beads revealed diffraction peaks in angles of 30.1°(220), 35.7°(311), 43.3°(400), 53.9°(422), 57.5°(511), and 63.0°(440) for Fe_3_O_4,_ and the diffraction peaks of 24.13(012), 33.15(104), 35.61(110), and 54.8 (116) for Fe_2_O_3_ crystal(Mishjil et al. 2015; Yazirin et al. 2017). Moreover, XRD analysis showed that the presence of Ni ions could be in the form of NiFe_2_O_4_ with the diffraction peaks at 30.1 (220), 35.7(311), (37.24) 222, 35.68(422), 57.38(511), and 62.95(440) planes(Parishani et al. 2017) (Fig. 8).

## Discussion

The purification of recombinant proteins is the most complicated issue in the downstream processes of pharmaceutical protein production. Therefore, the improvement of selective purification methods based on affinity chromatography using Ni-NTA resin and His-Tag label can play a significant role in shortening the time and cost of producing larger quantities of pharmaceutical and industrial recombinant proteins(Rosano and Ceccarelli 2014; Zhou et al. 2021). In previous studies, it has been shown to purify His-Tagged proteins, the magnetic agent after synthesis was coated with different organic/inorganic linkers or coating materials to chelate to nickel, copper, zinc, and cobalt ions (For example multiple coatings of magnetic materials with SiO2 and CTMS in the study of Lee et al., coating of magnetic materials with SiO2-TED, SiO2-IDA or SiO2-(APTES-ECH-ID) in the study of Zeng et al.)(Zeng et al. 2020). This coating of nanoparticles, in addition to making the process of synthesis of magnetic beads difficult and complicated, can impose more cost and time on the process of synthesis of beads, and finally purification of recombinant proteins to the system. Therefore, in this research, polydopamine nanoparticles were utilized and synthesized as a suitable bead for chelating ions with an easy method and without the need for expensive equipment. The presence of catechol, and indole groups on the surface of polydopamine nanoparticles provides the ability to chelate directly divalent ions such as nickel and iron (without the need for any linker and coating material on the surface of nanoparticles). SEM images confirm the synthesis of polydopamine nanoparticles with a diameter of about 120–140 nm. In a study conducted by Yang et al., magnetic nanoparticles were coated with dopamine hydrochloride after synthesis by Solvo thermal method, and then nickel ions were chelated on them. The results showed that after coating them with polydopamine, these nanoparticles become irregular in terms of morphology, and their thickness increases to 250 nm(Yang et al. 2015), while, in our study, polydopamine nanoparticles are almost spherical and uniform. Also, these nanoparticles have a smaller diameter (120 nm), which can be due to the direct chelation of Fe^3+^ and Ni^2+^ ions on the surface of polydopamine NPs. The size distribution plot of PDA/Fe^3+^/Ni^2+^ (Fig. 2b) shows a narrow size distribution in the range of 200 to 220 nm. This difference in the diameter of particles measured by the DLS technique and SEM microscope can be due to the self-aggregation property in DLS, which is observed as particles with a larger size, while in the SEM image, the boundary between the particles is distinct and distinguishable. Chelation of the surface of synthesized polydopamine nanoparticles with Fe^3+^ and Ni^2+^ ions at different weight amounts (nickel ions mg in mg PDA) was confirmed using a double ICP-OES test, and the result showed that increasing the number of nickel ions from 0.4 to 1.4 weight ratio, the adsorption of Ni^2+^ ion on the surface of polydopamine NPs increases, and the highest adsorption rate of Ni^2+^ ions on the surface of polydopamine NPs is in the weight ratio 1.4. Also, the results showed that each milligram of polydopamine NPs chelated with Ni^2+^ ions in a weight ratio of 1.4 mg/mg (Ni^2+^: PDA), is able to absorb about 3.7 ± 0.001 mg of Fe^3+^ ions, which is almost similar to other weight ratios. The SDS-PAGE analysis of Hsp40 showed that the protein purified by PDA magnetic beads in this study has suitable purity and reusability. The eGFP purification in elution 1 and 2 was confirmed by western blot, and the results were compared with the commercial Ni-NTA column. The amount of purified eGFP in elution 2 was 138.83 mg/ml, which is almost equal to the protein purified by the commercial Ni-NTA column (108.28 µg/ ml). In a study by Wang et al., the result showed that the concentrations of target proteins in the 1–3 eluents were 0.207, 0.066, 0.123 mg/ml, respectively, that the mean of its value is similar to purified protein in our study. According to the reported results, the synthesis of PDA NPs and the chelation of Fe^3+^ and Ni^2+^ ions were confirmed. The result of protein purification by PDA bead magnetic demonstrates that magnetic beads based on PDA NPs can be a suitable choice for the purification of His-tagged proteins. ICP-OES analysis also confirmed stability and the existence of Ni^2+^ and Fe^3+^ ions on the surface of PDA NPs after 10 days, and Fig. 3 proves that the ions were successfully chelated to the material. PDA nanoparticles greatly increased chelation sites of Ni^2+^, and improved the coordination affinity of nanoparticles for binding with His-Tag proteins. The hysteresis loop (Fig. 4) revealed the magnetic behavior of PDA/Ni^2+^ at room temperature after coating with Fe^3+^. The saturated magnetization (Ms) of PDA magnetic beads was 35.42emu/g. This value is similar to magnetization of Fe_3_O_4_ (40.6 emu.g-1), and more than synthesized Fe_3_O_4_/MPS@PAA/NTA-Ni^2+^beads (10.2 emu·g − 1) in a study by Wang et al. The magnetization of Fe_3_O_4_/MPS@PAA/NTA-Ni^2+^ beads decreased obviously due to surface modification of Fe_3_O_4_. Therefore, these beads have great potential for application in biotechnology and can be considered efficient magnetic beads for protein purification.

## Data Availability

All data generated or analyzed during this study are available from the corresponding author upon reasonable request.

## Abbreviations

IMAC: Immobilized Metal Affinity Chromatography
MNP: Magnetic nanoparticles
eGFP: Enhanced Green Fluorescent Protein
NTA: Nitrilotriacetic Acid
PDA: Polydopamine
SEM: Scanning electron microscopy
DLS: Dynamic Light Scattering
PBS: Phosphate-buffered saline
VSM: Vibrating sample magnetometery
UV-vis: Ultraviolet-visible spectroscopy
